# Symbiotic Outcome Modified by the Diversification from 7 to over 700 Nodule-Specific Cysteine-Rich Peptides

**DOI:** 10.3390/genes11040348

**Published:** 2020-03-25

**Authors:** Proyash Roy, Mingkee Achom, Helen Wilkinson, Beatriz Lagunas, Miriam L. Gifford

**Affiliations:** 1 School of Life Sciences, Gibbet Hill Road, University of Warwick, Coventry CV4 7AL, UK; proyash.roy@du.ac.bd (P.R.); ma2292@cornell.edu (M.A.); Helen.Wilkinson@warwick.ac.uk (H.W.); B.Lagunas-Castan@warwick.ac.uk (B.L.); 2 Department of Genetic Engineering and Biotechnology, University of Dhaka, Dhaka 1205, Bangladesh; 3 Department of Plant Pathology and Plant-Microbe Biology, Cornell University, Ithaca, New York, NY 14853, USA

**Keywords:** symbiosis, legumes, small-secreted peptides, Nodule-Cysteine Rich (NCR) peptides, terminal differentiation, genome amplification, nodulation

## Abstract

Legume-rhizobium symbiosis represents one of the most successfully co-evolved mutualisms. Within nodules, the bacterial cells undergo distinct metabolic and morphological changes and differentiate into nitrogen-fixing bacteroids. Legumes in the inverted repeat lacking clade (IRLC) employ an array of defensin-like small secreted peptides (SSPs), known as nodule-specific cysteine-rich (NCR) peptides, to regulate bacteroid differentiation and activity. While most NCRs exhibit bactericidal effects in vitro, studies confirm that inside nodules they target the bacterial cell cycle and other cellular pathways to control and extend rhizobial differentiation into an irreversible (or terminal) state where the host gains control over bacteroids. While NCRs are well established as positive regulators of effective symbiosis, more recent findings also suggest that NCRs affect partner compatibility. The extent of bacterial differentiation has been linked to species-specific size and complexity of the NCR gene family that varies even among closely related species, suggesting a more recent origin of NCRs followed by rapid expansion in certain species. NCRs have diversified functionally, as well as in their expression patterns and responsiveness, likely driving further functional specialisation. In this review, we evaluate the functions of NCR peptides and their role as a driving force underlying the outcome of rhizobial symbiosis, where the plant is able to determine the outcome of rhizobial interaction in a temporal and spatial manner.

## 1. Inverted Repeat Lacking Clade Legumes Impose Terminal Bacteroid Differentiation

Legumes benefit from a symbiotic association with soil rhizobia that enables them to take up biologically usable nitrogen (N) fixed by the bacteria in exchange for nutrients and carbon resources. In a successful symbiotic relationship, endosymbiotic rhizobia are able to colonise plant roots and induce changes in the host, resulting in formation of a specialized root organ called a nodule. In nodules, symbiotic host cells become enlarged as they undergo repeated endoreduplications (multiple rounds of genome duplication without cytokinesis) to accommodate thousands of rhizobial cells [1,2]. Rhizobia that colonise the host cells become surrounded by a host-derived membrane, forming an organelle-like structure called the symbiosome. Within symbiosomes, rhizobia undergo metabolic changes, and in particular legumes, they also undergo morphological changes, resulting in differentiation into their symbiotic forms (bacteroids) that are able to fix atmospheric dinitrogen into a form that is biologically accessible for the plant [3,4].

**Figure 1:**
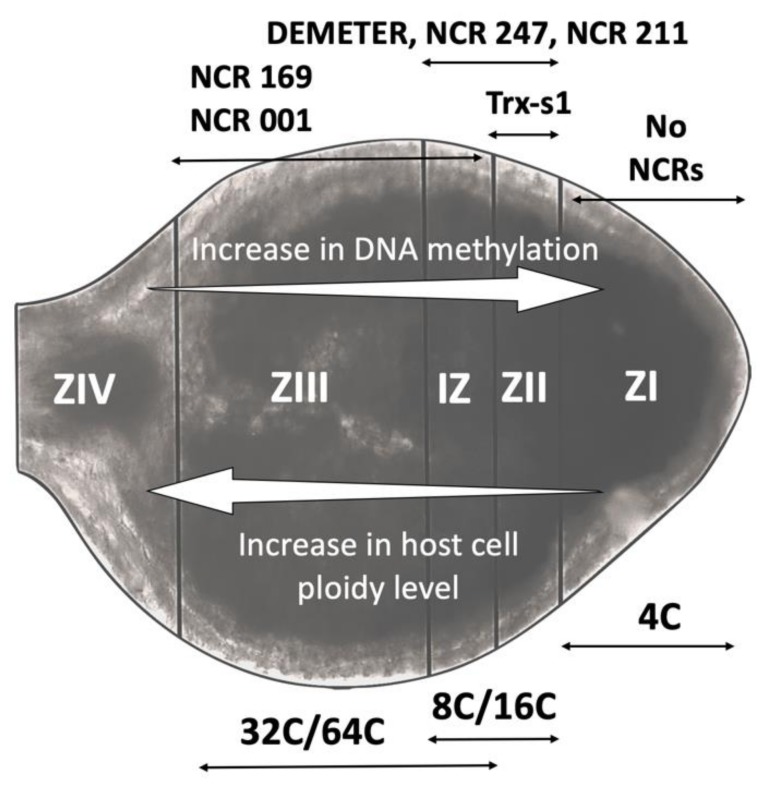
Schematic of the different zones of the nodule, their ploidy and location of nodule-specific cysteine-rich (NCR) expression. From the meristem (ZI) there are four distinct zones in indeterminate nodules whose ploidy level increases: the infection zone (ZII), the interzone (IZ), the nitrogen fixation zone (ZIII) and the senescence zone (ZIV). Zonal changes are also marked by specific expression of thioredoxin-s1 (Trx-s1) [9], the demethylase gene, DEMETER, and different levels of DNA methylation [10]. Transcriptomic analysis following laser-dissection capture of the different zones also suggest a varied and zone-specific expression of different NCRs [11,12]. The image in the drawing shows a nodule for illustrative purposes, with colour indicating levels of leghaemoglobin.

Depending on the legume host, nodules can be of different types in terms of their structure and development [5]. Legume species belonging to the Inverted Repeat Lacking Clade (IRLC), characterised by the lack of a 25-kb inverted DNA repeat in the plastid genome (e.g., *Pisum sativum*, *Medicago truncatula*, *Galega orientalis),* produce indeterminate nodules with a persistent meristem [6]. As reviewed by Pan et al. [7], indeterminate nodules can be spatially divided into successive developmental zones: the uninfected meristematic zone (ZI) that actively produces new nodule cells; the infection zone (ZII), where rhizobia infect host cells; the interzone (IZ), where both host and bacterial cells undergo profound changes and become significantly enlarged; the fixation zone (ZIII), where fully differentiated bacteroids fix nitrogen; and the senescence zone (ZIV), where both bacteroids and host cells die (Figure 1). Within indeterminate nodules of IRLC legumes, rhizobia are subjected to tight host control that results in terminal bacteroid differentiation (TBD). This is an irreversible change characterized by genome amplification, inhibition of cell division, cell elongation, increased membrane permeability and almost complete loss of bacterial reproductive ability [8]. Nodule functioning is different in non-IRLC legumes e.g., *Glycine max*, *Lotus japonicus*, *Phaseolus vulgaris.* Most non-IRLC species form determinate nodules that are round shaped and have short-lived meristematic activity. Bacteroids in determinate nodules do not show features of TBD and are capable of multiplying freely when isolated from nodules [5,7,8].

While endoreduplication occurs in both determinate and indeterminate nodules and results in symbiotic polyploid nodule cells (usually 64C or 128C, where C denotes haploid DNA content), rhizobial endoreduplication mostly occurs in the indeterminate nodules of IRLC legumes [8,13] and is comparatively rare or less significant outside the IRLC. For example, in *Medicago truncatula*, endoreduplication of *Sinorhizobium meliloti* results in an amplification of genome content up to 24C, compared with 1C/2C in free-living bacteria [8]. Interestingly, whilst the non-IRLC legume *Mimosa pudica* forms indeterminate nodules with a persistent meristem when infected by *Cupriavidus taiwanensis*, bacteroids are not terminally differentiated [14]. Therefore, TBD might not be related to nodule ontogeny or phenotype but rather to the genetic (e.g., IRLC vs. non-IRLC) and/or nodule-specific molecular (e.g., expression of certain group of peptides) profile of the host.

**Figure 2:**
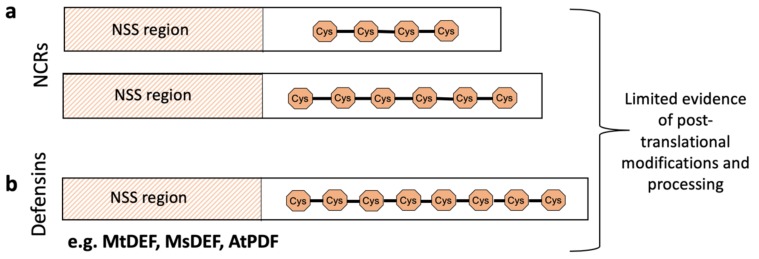
Simplified precursor structure of NCR peptides with defensins for comparison. (**a**) NCRs in indeterminate legumes such as *Medicago truncatula* and *Aeschynomene* are characterised by the presence of a signalling peptide at the 5’ end and four or six cysteines at conserved positions. (**b**) Defensins similarly have a 5’ signal peptide but have eight cysteine residues. Post-translational modifications, such as phosphorylation and lysine acetylation, have only been observed for eight NCR peptides: Medtr6g043380.1, Medtr7g051320.1, Medtr6g463200.1, Medtr7g029760.1, Medtr3g065710.1, Medtr2g044330.1, Medtr4g033290.1 and AES72906 [23].

Terminal bacteroid differentiation in IRLC legumes is mediated by a family of small peptides known as Nodule-specific Cysteine-Rich (NCR) peptides [15,16], that are expressed by the host almost exclusively in nodule cells [11]. All IRLC legumes analysed so far express NCRs, although the number of genes varies significantly, from 7 to ~700, depending on the host species [17]. As reviewed by Tavormina et al. [18], NCRs are cysteine-rich, defensin-like (DEFL) plant peptides that are derived from their non-functional precursors upon proteolytic cleavage of an N-terminal signal sequence (NSS). The NSS directs the peptide through the endoplasmic reticulum to the secretory pathway. NCR peptides are diverse and are only highly similar to each other in two regions—the NSS region [19] and four or six regularly spaced cysteine residues within the coding sequence (Figure 2a). Mature NCRs consist of around 20–50 amino acids including the four or six cysteines that potentially form two or three disulphide bridges. The number of cysteines distinguishes them from plant defensins that are usually 45–70 amino acid long and have eight cysteine residues (forming four disulphide bridges) (Figure 2b) [15,20,21]. Other distinguishing features between these small peptides are that defensins are expressed ubiquitously, not being nodule-specific, and are all cationic (isoelectric point, p*I*, ~ 9.0) [21]. In contrast, based on their p*I*, NCRs can be divided into- cationic, anionic and neutral [15]. Similar to defensins, certain cationic NCRs (e.g., NCR247, NCR335) exhibit strong in vitro antimicrobial activity [16,20]. However, among the NCRs detected inside bacteroids by Durgo et al. [22], most are anionic or neutral. The variety of expression location and differing molecular properties suggest that NCRs could play more diverse roles in vivo than the antimicrobial defensins.

To date, NCR peptides have not been reported outside IRLC legumes, with one exception of *Aeschynomene* spp., belonging to comparatively ancient dalbergoid lineage of legumes. The nodules of *Aeschynomene* spp. are neither determinate nor indeterminate; they have a central zone of infected tissue, which originates from the consecutive divisions of one or multiple cortical cells, initially infected though a non-transcellular, infection thread-independent process [24]. Therefore, these nodules do not have an uninfected primordium and both bacterial and host cell differentiate simultaneously from the start. However, they do express NCR-like peptides that are cys-rich and highly nodule-specific, and show signs of TBD, as bacteroids are enlarged, polyploid and irreversibly differentiated. Interestingly, NCR-like peptides in *Aeschynomene* spp. are different (in terms of sequence similarity) from their counterparts in IRLC legumes, suggesting two independent evolutionary events with convergent and common roles in regulating bacteroids [25]. 

TBD is likely to be beneficial for host legumes for a few reasons—firstly, it has been shown by Oono et al. [26] that swollen bacteroids are better at conferring host benefits (enhanced N-fixation and better plant growth per unit nodule mass) compared to their non-swollen counterparts in different host species. Therefore, terminally differentiated and elongated bacteroids could be considered to be more useful for their hosts. Secondly, the loss of bacterial reproductive capability could be a way to control bacterial number with the consequence of a better cost-gain trade-off for the host. Finally, as reviewed by Mergaert et al. [27], activity of symbiotic AMPs, such as NCRs, can result in introduction of pores or an increase permeability of bacterial membrane that might allow better metabolic exchange between partners. To what extent, if any, the terminal differentiation process offers an advantage over non-NCR expressing, non-IRLC legumes is not yet known, but if understood, it would be highly relevant for understanding legume-rhizobial co-evolution. In this review, we will focus on the current understanding of NCRs in terms of their role in TBD, their evolution and finally rhizobial molecular responses to NCRs. Much of our understanding of NCRs is based on the studies in the model legume *Medicago truncatula* and this will be our focus. Therefore, unless otherwise mentioned, any example or data presented in this review should be considered as representative of *M. truncatula*.

## 2. NCRs Primarily, But Not Only, Act as Mediators of Terminal Bacteroid Differentiation

A number of findings have implicated NCRs as the key mediators of terminal or irreversible bacteroid differentiation and amplification of their genome. Van de Velde et al. (2010) [16], ectopically expressed eight different *M. truncatula* NCR genes in *Lotus japonicus*, a NCR-lacking legume and reported that at least one of the them, NCR035, resulted in bacteroids with enhanced membrane permeability, cell elongation and compromised reproductive ability; all reminiscent of TBD. Moreover, in vitro treatment of free-living *S. meliloti* with a sub-lethal concentration of certain NCR peptides inhibited cell division and induced bacteroid-like characteristics [16,28]. In general, the extent of bacteroid differentiation depends on the size and complexity of the NCR gene family. A comparative study amongst ten IRLC legumes showed that the higher the number of expressed NCRs, the greater the degree of bacteroid elongation [17]. The extent of bacteroid elongation also correlates with their morphotype (shape), varying from swollen to elongated and elongated-branched, depending on the NCR profile of host species [17,29].

Particular NCRs have precise and non-redundant functions in different phases of rhizobial differentiation [30,31]. Two *M. truncatula* mutants, *dnf7* and *dnf4* (Defective in Nitrogen Fixation 7 and 4) that are disrupted in NCR169 and NCR211 functions, respectively, have a similar phenotype, with small and white nodules incapable of fixing nitrogen [30,31,32]. The transcriptomic profiles of both mutants are also very similar [32]. In wild type plants, both NCR169 and NCR211 share an overlapping expression pattern, where NCR211 is largely expressed in the infection and interzone and NCR169 is exclusively expressed in the interzone and mature fixation zone (Figure 1) [30,31]. Moreover, both NCRs localise to the peribacteroid space, the region between symbiosome membrane and bacteroids [7]. However, the extent of bacteroid differentiation is different in these mutants. While bacteroids in *dnf7* mutants (without NCR169) never reach terminal differentiation, bacteroids in *dnf4* mutants (without NCR211) die prematurely in the fixation zone just before or soon after reaching terminal differentiation [30,31]. These observations suggest that NCR169 has a critical role in terminal differentiation while NCR211 might be important for survival of differentiated bacteroids in the symbiosome. In both *dnf7* and *dnf4* mutants, bacteria do differentiate to some extent, therefore it is unlikely that NCR169 and NCR211 interfere in the cell cycle, unlike NCR247, discussed in Section 4. Moreover, their function might not be the elimination of incompatible bacteria because they are required for bacteroid survival and TBD. A pull-down assay suggests that NCR169 (as well as NCR28 and NCR290) might physically interact with NCR247, a cationic NCR with antimicrobial effect and unusually high p*I* (10.39) [33]. As discussed by Mergaert [34], some NCRs could function counteractively by binding one another to serve a precise function or to compensate or reduce the harmful effects of other NCRs. One hypothesis is that anionic or neutral NCRs might bind to cationic NCRs to antagonize their antimicrobial activity.

Proteins involved in the maturation process of NCR peptides are also important for effective symbiosis. This is illustrated in the case of *M. truncatula dnf1* mutants where a component of the nodule-specific peptidase complex Defective in Nitrogen Fixation 1 (DNF1), that cleaves the NSS from pre-mature NCR peptides, is non-functional. In the absence of functional DNF1, the NCRs that were tested by the authors were found to be trapped inside the endoplasmic reticulum, effectively blocking bacteroid differentiation [16]. It has been found that different plant- and bacteria-derived thioredoxin (Trx) and glutaredoxin (Grx) systems are necessary for nitrogen-fixing symbiosis (reviewed in [35]). For example, one plant-derived thioredoxin (Trx-s1) has been found to be induced in the nodule infection zone and to interact with several NCR peptides. While inactivation of Trx-s1 in the host plant negatively affects TBD, the ectopic expression of this enzyme in the endosymbiont partially enhances TBD in Trx-s1 inactivated plants [9]. It is likely that these redox enzymes play a crucial role in maintaining the appropriate cellular redox state including that of the multiple cysteine residues in NCR peptides, thus regulating their functions. Therefore, not only NCRs, but also other cellular components that regulate NCR activity, are essential for effective symbiosis.

## 3. Rapid Gene Family Expansion and Variation in Spatio-Temporal Expression Patterns Enable NCR Functional Diversity

The genome structure of the Papilionoideae subfamily that contains most nodulating legume species was strongly shaped by a whole genome duplication event around 58 million years ago (MYA) [36]. The origin of the IRLC group can be dated back to ~39 MYA, but exactly when and how the NCR gene family appeared is still far from clear [36,37]. One hypothesis suggests a recent origin of NCR genes that then rapidly expanded (possibly by gene duplication) and diversified in certain species (e.g., *M. truncatula*) [17]. This is supported by several observations—firstly, NCRs or genes that resemble NCRs from the IRLC and dalbergoid clades, respectively, are lineage specific (orphan) genes that lack orthologs, even in other closely related lineages [15,25]; this suggests an evolutionary recent origin. As NCRs are antimicrobial peptide (AMP)-like, and many AMPs are orphan genes like NCRs, it could be that NCRs evolved from a primitive AMP gene upon duplication and rearrangement. Secondly, NCR numbers vary highly (7 to ~700) among IRLC legumes, implying species-specific expansion. Mature NCR peptides from orthologous genes of different *M. truncatula* accessions contain signatures of both purifying (conservation) and diversifying selection, where other DEFLs are under purifying selection only [19]. NCR gene loci that are under purifying selection might share common functions, whereas those under diversifying selection could be rapidly evolving towards novel functions. This might be a good example of subneofunctionalization [38], where members of duplicated gene families could share and acquire new functions simultaneously. Finally, *M. truncatula*, which encodes over 700 NCR peptides (the highest number currently known), harbours relatively more locally duplicated genes (e.g., 3.1 fold higher than soybean) and a high number of transposons, located close to many NCR genes [10,36]. NCR genes are spread over the *M. truncatula* genome, often in small clusters [39]. Together, this genomic landscape suggests a process of transposon-mediated long-distance duplication, followed by local duplication and diversification of NCRs in this species. Such duplication events, might have affected, not only NCRs, but also other symbiosis-related gene families (e.g., leghaemoglobins, flavonoid signalling genes etc.) in *M. truncatula*, as their numbers are particularly high [36]. Among these symbiosis related gene families, some are also strongly linked to nodule function, e.g., Glycine Rich Peptides (GRPs) and Calmodulin-like proteins (CaMLs) [40]. In general, all of these genes are found in clusters across the genome, similar to with NCRs, suggesting duplication events was associated with their evolution [36,39,40].

The expression of many NCR genes are thought to be affected by DNA demethylation and histone modification [10,41]. In particular, one DNA demethylase gene, DEMETER (DME) was found to be expressed preferentially in differentiating nodule cells (Figure 1) [10], possibly involved in the activation of many NCRs. Moreover, activation of some NCR genes is associated with reduction in the suppressive histone (H) mark H3K27me3 (trimethylation of H3 lysine-27) and increase in the activating H3K9ac (acetylation of H3 lysine-9) [41], which could be part of the same methylation regulatory mechanism. As mentioned earlier, many NCRs are neighboured by transposons that are usually silenced by DNA methylation. Interestingly, DNA demethylation that activates NCRs can also result in activation of these transposons [10]. Therefore, these epigenetic modifications might have a role in the evolution or expansion in expression and functional diversity of NCRs. Our understanding of these evolutionary events should increase with the whole genome sequencing and molecular characterisation of additional IRLC legumes.

The functional diversity of NCRs is further highlighted by the observation that not all NCRs are expressed at the same time. In fact, different NCRs are activated in successive but overlapping waves, in both spatial and temporal manners, in different nodule zones during development [11,12,23]. A study taking advantage of laser capture microdissection coupled to RNA sequencing showed that only a few NCRs were activated in the distal part of the ZII (infection site) whereas others were activated in the proximal part of the ZII as well as in the IZ, and the ZIII [12]. Peak activation was observed at the IZ (with 411 activated NCRs), where bacteroids undergo rapid differentiation. No NCRs were detected in ZI and NCR activation was minimal in the ZIII (fixation zone) [11,12]. These findings suggest that NCRs are mostly activated by invading rhizobia and deactivated when bacterial differentiation is complete, supporting their role in TBD. Marx et al. [23] collected nodule samples at 10, 14 and 28 days post rhizobial inoculation and upon proteomic analysis on respective samples, they divided NCRs into early, intermediate and late, based on their relative temporal abundance. Early stage NCRs might be involved in enhancing rhizobial membrane permeability that allows other NCRs to enter the bacterial cytosol. Intermediate and late NCRs could target rhizobial metabolism to advance differentiation into N-fixing bacteroids [23]. Although NCRs are generally thought to be specific to nodules, at least two NCRs (NCR122 & 218) were reported to be expressed at a similar level in both nodules and uninfected roots, and three others (NCR077, NCR235 and NCR247) had shown somewhat reduced specificity [11], implying they might play other roles beyond that of the regulation of nodulation.

## 4. NCR Activity Targets the Rhizobial Cell Cycle and Other Crucial Rhizobial Cellular Pathways

**Figure 3:**
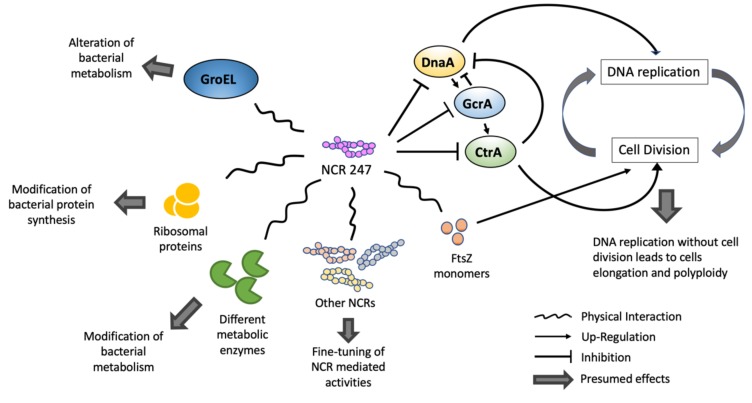
NCR247 modulates rhizobial activity via regulation of bacterial DNA replication, protein synthesis and metabolism. NCR247 appears to modulate a wide range of microbial pathways via directly repressing or binding to a range of bacterial proteins as well as other plant NCRs. It is possible that other yet to be characterized NCRs play similarly wide-ranging roles, supporting the diverse and important regulatory functions of NCRs during rhizobial nodulation.

Much of the proposed mechanism by which NCRs regulate bacterial activity is informed by the study of NCR247 [28,33], the best characterized NCR so far (Figure 3). The general proposition is that NCR peptides alter rhizobial cell cycle progression by targeting cell cycle components [34]. One such regulator is the CtrA protein that acts late in the cell cycle to repress replication initiation and promote cell division [42,43]. The loss of CtrA function is associated with the formation of swollen, polyploid bacteria that resembles bacteroids inside nodules. Therefore, it is possible that NCR peptides exert control by limiting CtrA function [43]. In vitro application of NCR247 downregulates *ctrA* gene expression, as well as most CtrA-regulated genes in *S. meliloti* [28]. Moreover, *ctrA* transcripts levels drop steadily along with bacterial differentiation [12], and in mature bacteroids the CtrA protein is almost absent [44]. In addition, mutations in bacterial genes that inhibit CtrA (e.g., *cbrA, cpdR or divJ*) result in abnormal bacteroid differentiation, leading to formation of non-functional nodules [34,44]. While all of these findings implicate CtrA as a potential regulatory node for NCR mediated effects, the actual mode of action is still not clear. NCR247 not only affects CtrA but also inhibits the expression of two other important rhizobial cell cycle regulators, DnaA and GcrA. DnaA is important for the initiation of prokaryotic DNA replication and it activates GcrA. Once activated, GcrA represses DnaA and upregulates CtrA to facilitate cell division [28,42]. While inhibition of GcrA (an activator of CtrA) fits the proposed mechanism, forcing the bacteroid to stop cell division, the presumed downregulation of DnaA is not so straightforward to explain, as the continuation of DNA replication is required for TBD. However, a study in *Caulobacter crescentus* suggests that CtrA and DnaA both compete for binding to the origin of replication to repress or initiate DNA replication, respectively [45]. Therefore, it could be that when CtrA levels are significantly reduced, even low levels of DnaA can still initiate DNA replication, enabling endoreduplication to take place. At the same time, DnaA activity is also regulated post-transcriptionally [28,42]; therefore, a lower transcript level does not necessarily represent a lower protein level or reduced activity. NCR247 is also known to interact with another conserved bacterial cell cycle protein, FtsZ [33], which is involved in the formation of the Z-ring and subsequent cell division [46,47]. NCR247 is thought to disrupt septum specific localization of FtsZ protein through inhibiting the polymerisation of FtsZ by binding to its monomers [28,33].

A number of other bacteroid cellular components have been found to interact physically with NCR247, including the chaperonin GroEL, ribosomal proteins, ATP synthase subunits, members of the nitrogenase complex and several TCA-cycle related enzymes [33,34]. The NCR247-GroEL interaction is particularly interesting as one of the five *S. meliloti groEL* genes (*groEL1*) is essential for functional symbiosis [48]. GroEL, in general, interacts with hundreds of proteins, facilitating their proper folding, thus binding might modify the NCR247 interaction network and overall function to severely alter bacterial metabolism. The interactions of NCR247 with ribosomal proteins and metabolic enzymes could be a means to modulate bacterial protein synthesis and metabolic activity, respectively [28]. It can be predicted that other NCRs also affect bacterial cellular pathways and have similarly broad effects. However, functional characterisation of other NCRs is needed to understand to what extent this is a widespread regulatory role, particularly since, unlike most other NCRs, NCR247 is not only nodule-specific, having low levels of expression in roots and stems. Therefore, NCR247 could have functions outside nodulation and might not be fully representative.

## 5. The Role of NCRs in the Legume-Rhizobia Arms Race

Legume-rhizobia symbiosis has mostly been depicted as an altruistic mutualism between two partners. However, a closer inspection of NCR-mediated TBD of rhizobia reveals a ‘scuffling’ situation where IRLC legumes subjugate their bacterial partner into a semi-viable, sterile state. To counter this, endosymbionts also upregulate different stress and defence related genes to minimize or escape from host-imposed controls (discussed in Section 5.2). Potentially, the higher the number of NCRs and the more diverse the NCRs are in the host, the greater the control over symbionts. However, ultimately, the outcome of the nitrogen fixation symbiosis depends on the host legume-endosymbiont balance to control each other’s functions.

So far, one observation suggests some rhizobial strains might have evolved mechanisms to avoid terminal differentiation entirely when interacting with IRLC legumes. *Glycyrrhiza uralensis* (liquorice), an IRLC legume able to trigger terminal differentiation with some rhizobial species (e.g., *Rhizobium galegae* bv. orientalis) [17], fails to enforce terminal differentiation when infected by *Sinorhizobium fredii* strain HH103 [49]. Although HH103 escapes terminal differentiation, it still fixes nitrogen for liquorice, resembling non-IRLC legume bacteroids. Remarkably, *S. fredii* HH103 also shows no or minimal sensitivity when treated in vitro with NCR247 and NCR335. HH103 bacteroids isolated from liquorice exhibit altered lipopolysaccharide (LPS) content on their outer membrane, a key molecule in legume-rhizobia interactions [50]. However, when HH103 infects non-IRLC legumes, *Glycine max* or *Cajanus cajan*, such modification of LPS was not observed [49]. LPS-modifications could therefore be a possible bacterial defence mechanism against NCR-mediated control. Furthermore, although *G. uralensis* expresses NCR peptides, only seven have been found to date [17], suggesting it might not have the genomic potential to ‘dominate’ over a broad range of rhizobial species. In many cases (discussed in the Section 5.1), counter-active measures between host and rhizobia result in incompatibility, even after successful initiation of nodulation. Therefore, in the arms race between legume and rhizobia, a fine-tuned balance has to be met in order to initiate and maintain functional N-fixing symbiosis.

### 5.1. NCRs Can Act as Determinants of Host-Symbiont Compatibility

**Figure 4:**
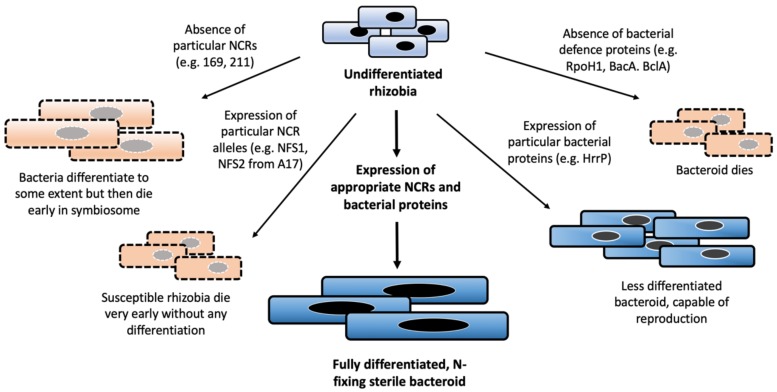
Expression of particular plant NCRs and bacterial proteins alters the fate of rhizobial bacteroids in the nodule. Research has identified the importance of NCRs not only that have defensin-like roles to kill rhizobia (e.g., NFS1 and NFS2 in *M. truncatula* A17) but that are required for bacterial survival and differentiation (e.g., NCR169, NCR211). Bacterial-expressed genes and defence proteins are similarly required for proper differentiation and bacterial survival, but also their capability to reproduce. When all required plant and bacterial proteins are expressed, rhizobia fully differentiate and are capable of nitrogen fixation.

Early interaction and symbiotic compatibility between legumes and rhizobia is first informed by the initial perception of rhizobial nodulation (Nod) factors by the host through its Nod-factor receptors [51,52]. Host legumes also recognise other bacterial effectors or surface proteins, e.g., LPS or microbe associated molecular patterns (MAMPs), and elicit immune responses against prospective symbionts. The success of symbiotic interactions depends on the extent of these immune responses and rhizobial susceptibility to them [53]. However, once these initial checkpoints have been successfully passed, symbiotic incompatibility can still occur at the later phases of nodule development and NCR peptides might play an important role at those points. Insight into this came from the discovery of two NCR-encoding genes, *nfs1 (*Nitrogen Fixation Specificity 1*)* and *nfs2*, that kill or do not kill infecting specific-strains of rhizobia, depending on the allelic variation of these genes. This has been demonstrated in two *M. truncatula* accessions, Jemalong A17 and DZA315.16, that, respectively, produce non-N-fixing (Fix-) or N-fixing (Fix+) nodules, with particular *S. meliloti* strains (A145 and Rm41) [54]. Further studies have revealed that A17 *nfs1* and *nfs2* alleles cause early senescence of bacteroids, whereas DZA315.16 alleles do not have such effect. The A17 alleles also act dominantly over DZA315.16 alleles in heterozygous plants, suggesting that A17 alleles might encode for peptides that kill those particular rhizobial strains, resulting in an incompatible interaction. However, other *S. meliloti* strains are not susceptible to *NFS1* and *NFS2*-mediated killing, regardless of which alleles are present. Other than this strain-specific antimicrobial effect, NFS1 and NFS2 might not be essential for N-fixing symbiosis since elimination of the genes altogether results in functional symbiosis [54,55,56]. The sensitivity of particular bacterial strains to NFS1 and NFS2 might be due to the chemical structure of the bacterial exopolysaccharides (EPS). Interestingly, the EPS of sensitive strains are significantly less succinylated than the EPS of the compatible strains. Moreover, expression of EPS compatible strain biosynthesis genes in an incompatible strain resulted in compatibility [57,58]. Therefore, the presence of abundant negatively charged succinate groups in the bacterial EPS of the compatible strains (along with alteration in LPS, as discussed earlier) could act as a protection mechanism against the antimicrobial effect of particular NCR peptides, potentially disrupting their recognition or binding. This idea is further supported by the finding that expression of exopolysaccharides in *S. meliloti* is associated with better protection from NCR247 [59]. Nonetheless, the example of *nfs1* and *nfs2* highlights one mechanism for how host NCRs dictate the outcome of symbiotic interactions (Figure 4).

### 5.2. Rhizobia Fight Back! Bacterial Defence Against NCR Action

A number of rhizobial stress-related genes are upregulated when *S. meliloti* is treated with NCR247 or NCR335. Most prominent among them are: an RNA polymerase σ-factor, RpoH1; a heat shock protein, IbpA and a methionine sulfoxide reductase A, MsrA1 [60]. Upregulation of RpoH1 is particularly interesting as *rpoH1* mutants die prematurely in the symbiosome, reflecting that RpoH1-related responses are important for bacteroid survival [61]. Since host plant cells infected by rhizobia introduce various oxidative/nitrosative stresses to bacteria [62], the function of an antioxidant enzyme, such as MsrA [63], and other rhizobial catalases and superoxide dismutases seems important for proper bacteroid differentiation [64]. The two-component regulatory systems ExoS-ChvI and FeuP-FeuQ, and their regulons that are involved in EPS and cyclic glucan production, respectively [65,66], are also rapidly induced in rhizobia upon treatment with NCR247 [28]. Many pathogens also use two-component systems to sense AMP activity [67] and rhizobial mutants disrupted in ExoS-ChvI and FeuP-FeuQ functions fail to promote infection thread development and functional symbiosis [65,68]. Such observations suggest that these two-component systems might be utilized by rhizobia to sense and promote resistance against NCR peptides in nodules.

Apart from these broad-spectrum stress-related responses, rhizobia have some unique strategies that seem to enable them to counteract the effects of NCRs. For example, two orthologous membrane transporter proteins, BacA in *S. meliloti* and BclA from *Bradyrhizobium spp*., are critical for symbiosis in NCR-producing legumes [69,70,71]. *bacA* and *bclA* bacterial mutants initiate nodulation normally but die early in the symbiosome of NCR-producing legumes. It has been found that *bacA* mutants persist longer inside nodules of *M. truncatula* mutants deficient in NCR functions (e.g., *dnf1*) [70]. BacA and BclA are similar to ABC transporters and are involved in the import of a large spectrum of peptides [72]. Therefore, it is hypothesised that these transporters act by importing NCR peptides into the cytosol, effectively removing them from the cell surface (the site of action for many AMPs). Since the homologs of *bacA* and *bclA* are widely present in all rhizobia, and even in some non-symbiotic bacteria [34,69,71], it is possible that their role in symbiosis might be supplementary to their unique non-symbiotic functions. In addition, because the presence or absence of these transporters determines whether symbionts would be able to persist in certain legumes, these transporters might have played an important role in symbiotic co-evolution, in determination of legume-rhizobia partner specificities.

Some rhizobia might have evolved a more efficient way of resisting NCRs. *S. meliloti* strain B800, carrying an accessory plasmid (pHRB800), has been found to produce Fix+ nodules in *M. truncatula* accession A17, but Fix- nodules in accession A20 [73]. A closer inspection reveals that the plasmid contains an M16A family zinc-metallopeptidase-encoding gene that is capable of degrading many NCRs of different p*I*, in vitro, albeit with varying efficiency. This gene while present in rhizobia affects N-fixation with several *M. truncatula* accessions, but the same bacterial strain without the gene has no effect on N-fixation with corresponding hosts. Therefore, the gene is designated as a host range restriction peptidase (*hrrP*) as its presence limits the effective N-fixing host range of rhizobia. Interestingly, HrrP-expressing (HrrP+) bacteria proliferate better in both functional (A17 accession) and non-functional (A20 accession) *M. truncatula* nodules compared to *hrrP* mutants. This is likely because HrrP diminishes the host NCR arsenal completely or to some extent. It is, however, not clear whether NCRs are the natural substrate of HrrP or if it simply degrades many NCRs, as they are analogous to the so far ‘unknown’ substrate(s). It is also not clear how some hosts (e.g., A17) induce HrrP+ bacteria to fix nitrogen to a normal level [74]. Variability of NCR genes among different *M. truncatula* accessions [19] and possible differences in their expression patterns could be an answer to this. Nevertheless, from the perspective of an individual bacterium, having *hrrP* is an advantage, no matter whether they fix nitrogen or not, since their reproductive capability is less compromised compared to HrrP-lacking strains [7]. Phylogenetic analysis has revealed that some distantly related *Sinorhizobium* isolates contain very similar *hrrP* alleles, while some closely related strains have comparatively divergent *hrrP* sequences. Interestingly, *hrrP* sequences are surrounded by transposable elements that are likely to be from the genus *Rhizobium* in origin. Moreover, various *Rhizobium* species harbour *hrrP* homologs in their chromosomes. Therefore, it is proposed that *Sinorhizobium* might have acquired *hrrP* from *Rhizobium* through a relatively recent horizontal gene transfer event, and then the gene has spread among various *Sinorhizobium* strains [74], being selected within these strains for its positive benefits for the bacteria.

## 6. Discussion and Perspectives

Comparative study [17,29] and examples from specific species suggest that legumes with a higher number of NCRs apply tighter control mechanisms over bacteroid metabolism, becoming more dominant over them. This could be because: firstly, a concoction of many NCR where some sharing similar functions and others with *de novo* functions, add robustness in the system; secondly, diversified NCRs are likely to be more effective against bacterial defence (e.g., HrrP would not be equally efficient for NCRs with varying p*I*). While the host benefit from bacterial TBD is quite obvious, how bacterial partners benefit from TBD is not yet clear. It could be that the subset of bacteria that are proliferating in the infection thread or those saprophytic bacteria at the proximal region of senescence zone (mentioned by Timmers et al. [75]), contribute significantly to the bacterial population and their natural persistence from an evolutionary perspective. Alternatively, perhaps the apparent submissive status of differentiated bacteroids was also adopted by the bacterial predecessors during endosymbiosis (e.g., mitochondria, chloroplast) [76]. It could be that the rhizobia-host symbiotic interactions are a part of an early evolutionary stage of a future plant N-fixing organ. We can speculate that the rapid expansion of the NCR gene family, therefore, might be part of an evolutionary strategy between species to domesticate their bacterial partners to a status where the acquisition of a new stable endosymbiosis is possible. Some support for this hypothesis comes from study of the amoeba *Paulinella chromatophora,* which contains an evolutionary early stage photosynthetic organelle called a chromatophore (derived from cyanobacterium approximately ~100 MYA). It has been shown that *P. chromatophora* directs a number of small peptides that are <90 amino acid long (along with other larger proteins) to chromatophores. These peptides mostly come from the ancestral host (not from ancestral cyanobacteria); thus, are likely to play vital role during early stages of chromatophore integration [77]. Of course, such integration of endosymbionts would be much complicated in multicellular organisms compared to single cell amoeba, but something similar has happened in the case of aphids-*Buchnera (*discussed below). 

Defensin-like peptides with symbiotic functions are evolutionary extraordinary but not unique to legume-rhizobia interactions. *Alnus glutinosa*, an actinorhizal species that forms N-fixing symbiosis with the actinobacterium *Frankia*, expresses such peptides in their nodules. One of those peptides (Ag5) has been found to induce profound physiological changes in *Frankia* with increased membrane permeability [78]. Such symbiotic peptides were also found outside plants, for example, in the endosymbiosis between aphids and their bacterial endosymbiont *Buchnera aphidicola.* The Aphids-*Buchnera* symbiosis was established at least 200 MYA, and has now reached a state of obligate mutualism where Buchnera no longer lives freely outside the host and these γ-proteobacteria are vertically and maternally transferred through host generations [79]. The aphids harbour endosymbionts in specialized cells called bacteriocytes where at least seven cysteine-rich peptides were found to be expressed [79,80]. Although the exact functions of these bacteriocyte specific cysteine-rich (termed BCR) peptides are still unknown, their bacteriocyte-specific expression pattern suggests a role in regulating the symbiosis.

As outlined above, there is still much we need to know about NCRs, but their presence in different independently evolved cases of symbiosis suggests that they share common and important symbiotic functions. Since NCRs or NCR-like peptides show highly tissue type-specific expression and the expression level can be also modified by different factors (e.g., nitrate provision or phosphorus (P) deficiency can result in downregulation of NCR genes [81,82]), studies investigating how their activity varies between different cell types and environmental conditions would be highly informative. In that regard, identification and characterisation of transcription factors that are involved in the nodulation process and possibly in the regulation of NCRs (e.g., the ethylene response factor required for nodule differentiation, abbreviated as EFD [83]) would be very useful. In a previous study [84], five conserved motifs were identified in the promoter region of *M. truncatula* NCRs, but further studies focusing on their molecular interactions (e.g., between putative *cis-* & *trans-* regulatory elements) will shed light on the NCR regulatory networks. As certain NCRs are antimicrobial in vitro (including the symbiotically indispensable NCR211), an interesting hypothesis [85] is that aside from their symbiotic roles, NCRs confer a general immune protection to nodules by preventing entry of undesirable microorganisms, at the same time as preventing symbiont overgrowth. These suggestions, building on our current knowledge, highlight how complex the scenario likely is within nodules. To understand how TBD and nitrogen fixation are controlled by host and symbiont, the understanding of both legume and rhizobial genomes is crucial. The structural and functional characterisation of different symbiotic peptides from different symbiotic systems will certainly improve our understanding in this regard. Such knowledge might, indirectly, also help us to develop novel therapeutics or agricultural products with better antimicrobial or pest control measures. With the advances in next-generation sequencing and omics techniques being more accessible in non-model plant and microbial species, we are moving closer to gaining a deeper understanding of rhizobial-legume symbiosis.
